# A new full-field XRF imaging station at Synchrotron Light Research Institute

**DOI:** 10.1107/S1600577523002722

**Published:** 2023-04-24

**Authors:** Wantana Klysubun, Pongjakr Tarawarakarn, Noppawath Thamsanong, Sompin Mahakhod, Penphitcha Amonpattaratkit, Chanakan Cholsuk

**Affiliations:** a Synchrotron Light Research Institute, 111 Moo 6 University Avenue, Muang, Nakhon Ratchasima 30000, Thailand; b 24-4 Achasan-ro 5-gil, Seongdong-gu, Seoul 04793, Republic of Korea; University of Essex, United Kingdom

**Keywords:** synchrotron-radiation-based full-field XRF imaging, Mn image of mineral dendrite, Sn and Cu dealloying in corroded bronze, Zn distribution in fish eye

## Abstract

A full-field X-ray fluorescence imaging (FXI) station was recently developed at beamline BL8 of Synchrotron Light Research Institute (SLRI), Thailand. An unfocused, synchrotron X-ray beam from the bending magnet with a size of 2 mm (vertical) × 13 mm (horizontal) and photon energy of 10 keV was employed in the FXI experiments. A sample stage was tilted by 7.5° to enlarge the vertical beam size. X-ray fluorescence images were recorded by an energy-dispersive, 256 × 256 array, pn-type charge coupled device detector equipped with a polycapillary optics, providing a full-frame image size of 12.3 mm × 12.3 mm. The incident photon flux per pixel was 3 × 10^4^ photons s^−1^ (100 mA)^−1^ and the experimental spatial resolution was 68 µm. Image processing was carried out offline using an in-house MATLAB program capable of elemental selection and inhomogeneity intensity correction. Elemental detection limits of FXI were found to decrease with increasing atomic number, *i.e.* 0.3 to 0.03 wt% for *Z* = 19 (K) to 30 (Zn). Compared with the BL6b microbeam imaging (µXI) station at SLRI with higher photon flux per pixel, 3 × 10^10^ photons s^−1^ (100 mA)^−1^, a tenfold sample area can be obtained and 13 times higher peak-to-background (PKB) ratio at Zn *K*_α_ measured with the same experimental time (8 h). Simultaneous measurement of FXI is more time-efficient against the long overhead times of µXI scanning over large pixel numbers, >65000. To demonstrate potential applications of the new FXI station, various types of samples were examined: dendritic limestone, ancient bronze and dried fish. Analyzed elemental images enabled us to identify areas rich in Mn on the limestone, Sn and Cu separation in the bronze, and Zn nutrition in the dried fish eye.

## Abstract 2

The development of a full-field X-ray fluorescence imaging technique using a large synchrotron X-ray beam at Synchrotron Light Research Institute (Nakhon Ratchasima, Thailand) is described. Various tests were conducted to determine experimental specifications and performance. Elemental distribution images are reported for dendritic limestone, corroded bronze and dried fish.

## 1. Introduction

X-ray fluorescence (XRF) imaging has become a well known analytical technique for its unique ability to reveal elemental distribution in materials with a typical micrometre-scale spatial resolution. Among other research fields, it has been frequently applied in biology, medical science, geology and archeological science. An extensive review of the technique applied on plants was presented by Kopittke *et al.* (2018 ▸), describing novel findings of elemental distributions relating to bio-molecular functions, food-product nutrients, foliar fertilization and toxic effects. For example, enrichment of micronutrient Fe, Cu and Zn minerals was found in alerone cells of wheat kernels (De Brier *et al.*, 2015 ▸). Similarly, abnormal accumulation of some metals such as Ni and Co in cancerous tissues has been revealed by X-ray fluorescence (XRF) imaging (Uo *et al.*, 2015 ▸), and high densities of Cu and Fe were reported in Wilson’s disease liver tissues (Hachmöller *et al.*, 2016 ▸). XRF imaging is also considered as a non-destructive tool making it suitable for historical art and archeological studies. To name a few, Dik *et al.* (2018 ▸) and Howard *et al.* (2012 ▸) and have mapped a yellow-pigment Sb in a Vincent van Gogh painting and a white-pigment Zn in a self-portrait by Arthur Streeton, respectively. In geology, inhomogeneity is a common characteristic of rocks and ores, and thus elemental mapping remarkably helps to characterize micro-scale chemical constituents, *e.g.* Si, K, Ca and Fe, being reported in different regions in granite (Flude *et al.*, 2017 ▸).

XRF imaging has been widely developed at large synchrotron facilities. Experiments are typically performed using a focused, monochromatic, synchrotron X-ray beam ranging in size from 100 µm down to 50 nm. Experimental data are obtained by moving a sample through the focused beam at a fine step (*i.e.* a pixel) over an area of interest (AOI). At each step an XRF spectrum is collected using an energy-dispersive X-ray detector, which is normally set at 90° to the incident beam and in the polarization plane to reduce elastic and inelastic scattering (Hanf *et al.*, 2016 ▸). A Maia detector is also a favorable choice because of its larger solid angle in back-scattering position geometry (Ryan *et al.*, 2014 ▸). Nonetheless, this micro-beam XRF imaging (µXI) approach is challenging for an AOI consisting of more than several tens of thousands of pixels as it requires a very fast dwell time (*e.g.* ms pixel^−1^), good signal-to-noise statistics and rapid scanning mechanics. Therefore, the µXI techniques are mostly available at high-flux synchrotron facilities. Regarding image processing, various computer freeware, such as *PyMCA* (Solé *et al.*, 2007 ▸), *Mantis* (Lerotic *et al.*, 2014 ▸) and *MMX-I* (Bergamaschi *et al.*, 2016 ▸), have been developed over the last decades.

Since 2011, the invention of a full-field X-ray detector has led to a novel approach to perform XRF imaging experiments. This detector is a pnCCD array detector, operated equivalently to an optical camera, which is what we use to take pictures; therefore, it is commercially known as a color X-ray camera (CXC) (Scharf *et al.*, 2011 ▸). As opposed to µXI, FXI employs large X-ray beams such as those generated by X-ray tubes or unfocused synchrotron X-ray beams. A sample is constantly irradiated, and the consequent XRF signal emitted from the sample is spatially resolved by a pinhole or polycapillary optics mounted in front of the detector (Romano *et al.*, 2013 ▸, 2016 ▸). Measurement time mainly depends on counting statistics.

In this work, we will describe a new FXI station using a large, monochromatic X-ray beam from beamline BL8 of Synchrotron Light Research Institute (SLRI), Thailand (Klysubun *et al.*, 2019 ▸). BL8 utilizes synchrotron radiation from a 1.44 T bending magnet of a 1.2 GeV synchrotron machine, delivering an unfocused monochromatic X-ray beam, which has been used for X-ray absorption spectroscopy (XAS) since 2006. The X-ray photon energy is tunable from 1 keV to 13 keV using a double-crystal monochromator equipped with interchangeable crystals: beryl(1010), KTP(011), InSb(111) and Ge(220). The X-ray beam is only collimated in the vertical direction by an Rh-coated Si mirror, yielding a large beam size of 13 mm (width) × 2 mm (height) at normal incidence. Since photon flux above 5 keV is gradually decreased with increased photon energy, the highest photon energy for FXI is 10 keV, at which the photon flux is 10^9^ photons s^−1^ (100 mA)^−1^. To evaluate the performance of our new FXI station, spatial resolution and detection limits were experimentally determined using various internal standards. In addition, experimental data obtained from the FXI station were compared with those from a µXI station at SLRI. Lastly, mineral dendrite, ancient bronze and small dried fish were measured to demonstrate possible applications of the FXI station.

## 2. Full-field XRF imaging station

**Figure 1:**
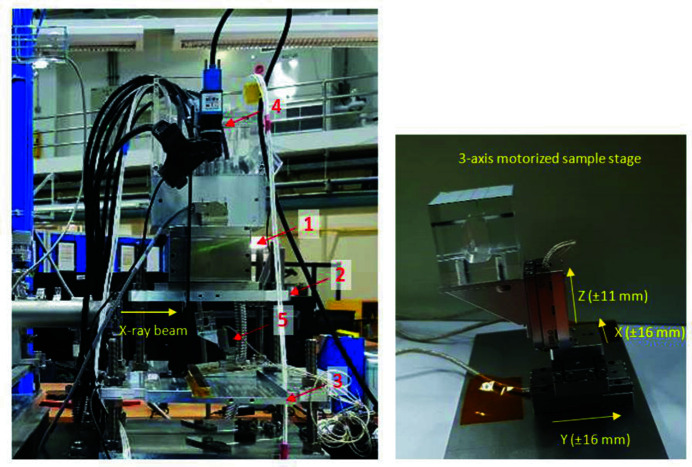
Photographs of the FXI station at the BL8 beamline, showing the color X-ray camera (1), top and bottom alignment tables (2) and (3), optical camera (4) and three-axis motorized sample stage (5).

Fig. 1 ▸ shows the new FXI station installed downstream of the BL8 XAS station where the X-ray beam was transferred to the new station via a long He-gas flow pipe terminated with a perforated polypropyl­ene window. The FXI main instruments include a CXC, cooling system, polycapillary optics, alignment tables, motorized sample stage and magnifying optical camera.

The CXC was manufactured by PNDetector GmbH, Germany; its first prototype was developed by Scharf *et al.* (2011 ▸). Table 1 ▸ provides technical specifications of this detector. Its full image area is 12.3 mm × 12.3 mm comprising 256 × 256 detector pixels. During operation, the CXC was evacuated at 0.13 mbar and then cooled down to −20°C by two thermoelectric modules. A Be window (50 µm thick) was mounted at the CXC entrance for vacuum protection. The parallel polycapillary optics (1:1 focusing ratio, Fig. S1 of the supporting information), with a 21 µm channel diameter and 37 mm length, was mounted in front of the Be window housing.

The CXC was mounted upside down on the top alignment table while the sample stage was on a bottom one. Both tables were independently adjustable in the horizontal (*X*) and vertical (*Z*) directions enabling the sample stage to be aligned with the X-ray beam and the camera with the sample stage. These two alignment steps were mandatory after repositioning of the incident X-ray beam, which could be done after changing the DCM crystals to Ge(220) and photon energy tuning at 10 keV for FXI experiments. The sample stage was driven by three miniature piezoelectric motors (Q-521, Physik Instrumente, Germany) so that a sample mounted on this stage could be moved in the *Z* direction for height adjustment (±11 mm) and in the transverse (*X*) and longitudinal (*Y*) directions for selecting an AOI frame (±16 mm) on the sample. The maximum weight of the sample was limited to 80 g due to the small load capacity of the *Z* motor. For the best spatial resolution, the distance from the capillary optics to the sample surface was set as short as possible (Ordavo *et al.*, 2011 ▸), *i.e.* 2 mm, by adjusting the *Z* motor. Since the incident beam size in the vertical direction is only 2 mm, the sample stage was necessarily tilted by 7.5° to the beam direction to extend the area of the beam on the sample. Therefore, the final size of the incident beam footprint on the sample was 13 mm × 15 mm. Samples with smooth, flat surfaces are most suitable for the inclined incidence as they are free from shadowing effects. Prior to a measurement, a photograph of the selected AOI area was taken using the optical camera (DFK 23UP031, The Image Source, Taiwan).

The CXC was operated in single-photon-counting (SPC) mode such that only single-photon events are allowed. In SPC mode, a photon can be analyzed regarding its position, time and energy. This is done by setting a frame rate at a high speed (200 Hz, 400 Hz or 1000 Hz) that is fast enough to allow less than one photon per pixel on average in one readout cycle, *e.g.* 5 ms for 200 Hz. In other words, each pixel is hit by one photon or not at all. Since the photon generates a charge cloud, which may spread between adjacent pixels (two to four pixels), its centroid position or the origin of the event is determined from the sum of the spread charge and then correspondingly assigned to the origin pixel. Roughly 1/40 of the pixel number in one frame can be used to estimate the maximum integral count rate (Leitenberger *et al.*, 2008 ▸), *i.e.* the input count rate; for example, at 200 Hz the maximum input rate is about 3 × 10^5^ counts per second (cps). Above this limit, the CXC operation results in pixels of false photon energies. For a normal Si(Li) detector, Martínez-Criado *et al.* (2012 ▸) reported a similar operation false occurred at a much lower input rate, *i.e.* 8 × 10^4^ cps, resulting in 30% count loss or dead-time. Therefore, the SPC mode of CXC can work much faster.

The photon energy is analyzed in arbitrary digital units (ADUs), ranging from 0 to 2000 and determined from the sum of split charge (Leitenberger *et al.*, 2008 ▸). Four gain values of 32, 64, 128 and 256 can be selected for measuring the photon energy from 0 up to 7, 14, 28 and 56 keV, respectively. An XRF spectrum was consequently recorded in every pixel and readout at the selected frame rate. All pixels in a frame were divided in half to be processed in two opposite directions using four multiplexing ASIC amplifiers. This resulted in fast readout up to a 1000 Hz frame rate. Summed up over pixels and frames, the CXC can be used in just the same way as a normal XRF detector.

The CXC system was controlled by a Linux-based computer installed with the *cosClientGUI* software for: operating the camera and thermoelectric modules; monitoring the camera temperature and vacuum status; and setting gain, frame rate and frame number. Experimental data were acquired using the *pyServ* software and stored as a 256 MB binary file. To create multi-element XRF images from the data file, a MATLAB program was developed in-house. It was used offline to convert the data binary file to a text file of 2000 rows (ADUs) × 65535 columns (detector pixels) and process the data as follows. The ADUs were converted into photon energies as described in Section 3.1. A full-frame XRF spectrum was then derived from the sum of photons (counts) over all pixels at every photon energy. For an observed element, a region of interest (ROI) was set over one standard deviation of its respective Gaussian peak of *K*_α_ or *L_α_* in the full-frame spectrum, and the number of photons in the ROI was integrated for each detector pixel and stored in its row and column. The final output was a 256 × 256 square matrix that could be graphically displayed as the elemental XRF image. Furthermore, the matrix could be exported and viewed by other applicable programs. A loop structure was used in the MATLAB program for processing XRF signals of other elements in the full-frame spectrum.

## 3. Performance tests

### 3.1. Photon energy calibration

A calibration sample (C1) was prepared from a mixture of CaO, KCl, TiO_2_, V_2_O_3_, Cr_2_O_3_, MnO, FeO, CoO, NiO, CuO and ZnO in amounts of 1 mmol each, *e.g.* 74.6 mg of KCl. The mixture was homogeneously ground using a small pulverizer and then pressed into a pellet. The C1 pellet was measured at the FXI station with a gain of 128, a frame rate of 200 Hz and frame number of 2.88 × 10^6^. The incident X-ray beam photon energy was 10 keV. In the C1 full-frame spectrum, it was found that *K*_α_ peak positions in ADUs linearly increased with theoretical photon energies of the *K*_α_ lines (Fig. S2). Consequently, the best fit was determined by linear regression, yielding the following photon energy calibration parameters: 7.26 eV ADU^−1^ (slope) and 43.5 eV (offset). The measured photon energy gives a very linear fit with the ADUs as shown by the almost ideal value of the adjusted *R* ^2^ value of the fit, *i.e.* *R* ^2^ = 0.99999.

### 3.2. Spatial resolution

The experimental spatial resolution of the FXI technique was determined using thin Cu foil (thickness 5 µm) and Cu wire (diameters 25 and 50 µm) pasted on polyimide tape such that their edges were lined up with the transversal and longitudinal directions of the CXC. This prepared specimen was measured for the same conditions as for C1, and its Cu image matrix was obtained using the MATLAB program described previously. Next, Cu foil data were integrated over longitudinal pixels (columns) and then plotted against transversal pixels (rows), yielding a step-like function. Taking the first derivative resulted in a Gaussian curve. The Gaussian peak position corresponded to the foil edge while the Gaussian width determined the transversal spatial resolution. Similarly, the longitudinal spatial resolution could be measured. An additional Gaussian curve was also derived from the Cu wire data and its fitted width was deconvoluted with the wire diameter to obtain the experimental spatial resolution. As shown in Table 2 ▸, all spatial resolution values obtained from the thin foil and wire are in good agreement, and the average spatial resolution was found to be 68 µm.

### 3.3. Detection limits

As presented in Table 3 ▸, the pixel detection limit (PDL) was determined from the C1 data. In a single pixel, the average net intensity (*n*) of K to Zn was overall increased from 2 × 10^−5^ to 104 × 10^−5^ cps while the average background (*b*) was much lower and similarly increased from 3 × 10^−8^ to 33 × 10^−8^ cps. We determined the PDL using the following equation (Jenkins & De Vries, 1970 ▸): PDL = 4.65(2*b/T*)^1/2^ *c*/(*n*−*b*). Here, *c* and *T* are the elemental concentration and measurement time(s), respectively. Hence, our PDL for K was 3.9 wt%, while it was more sensitive for Zn, *i.e.* 0.2 wt%. This is to be expected as X-ray transmission for air and Be windows increases with X-ray energy.

### 3.4. Inhomogeneity of the synchrotron X-ray beam

**Figure 2:**
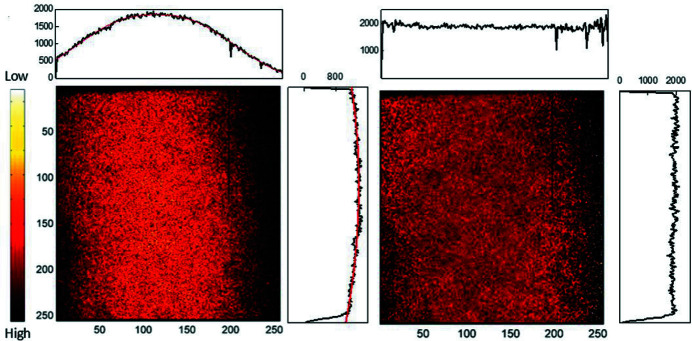
Images of the Cu plate before (*a*) and after (*b*) normalization. The red curves represent the best fits of the intensity profiles.

Synchrotron radiation produced by a bending magnet has a characteristic intensity profile; in the vertical direction it is theoretically described by a Gaussian function, whereas it is uniform in the horizontal direction (Mobilio *et al.*, 2015 ▸). Therefore, the 10 keV incident X-ray beam profile used in the FXI station was necessarily investigated using a homogeneous Cu plate. The experimental Cu image is shown in Fig. 2 ▸. Since the Cu plate was inclined by 7.5°, the longitudinal profile appeared as a pseudo-Voigt function while the transversal one was nearly flat. Considering this X-ray beam inhomogeneity, a normalization matrix (NM) was necessarily constructed by fitting the Cu plate data with pseudo-Voigt and polynomial functions. Finally, a sample image (S) could be normalized as S/NM. For validation purposes, the same Cu plate was also measured for a second time, and its normalized image (S/NM) is shown in Fig. 2 ▸, correctly representing a homogeneous Cu distribution of the Cu plate.

### 3.5. Comparison with micro-beam XRF imaging

**Figure 3:**
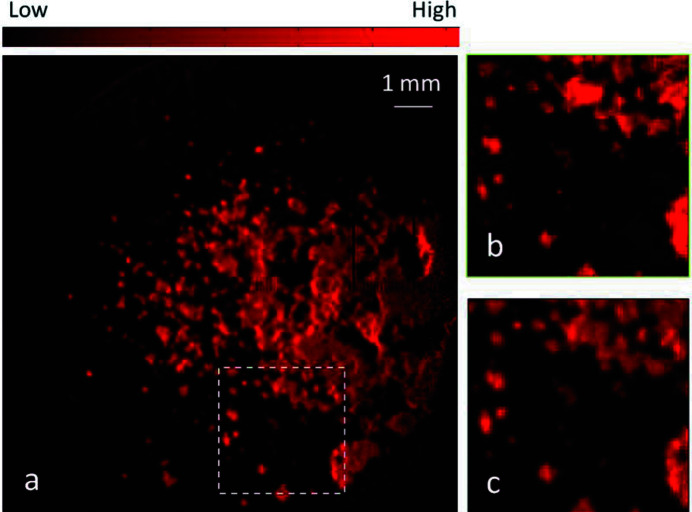
Zn image of the C2 sample recorded at the FXI station (*a*), and Zn image in the dashed frame recorded at the µXI (*b*) and FXI (*c*) stations.

A test sample (C2) was prepared using CaO, FeO and ZnO. To obtain a non-uniform pattern, each oxide was arbitrarily deposited and pressed on a boron nitride pellet (13 mm in diameter). The C2 sample was measured at the FXI station using a frame rate of 200 Hz, frame number of 5.76 Mf (where Mf means ×10^6^ frames), 8 h total exposure time and 3.9 × 10^8^ total incident photons per pixel. For comparison purposes, C2 was measured at the µXI station of the SLRI beamline BL6b (Muhd Amli *et al.*, 2020 ▸). The X-ray beam at BL6b is a micrometre-sized pink beam produced from an SLRI bending magnet, and its polychromatic photon flux decreases with photon energy: 2 × 10^12^ to 3 × 10^10^ photons s^−1^ (100 mA)^−1^ from 4 keV to 10 keV. The µXI detector was a four-element Si drift detector (with 12.5 µm-thick Be window). The experimental beam size and the scan step were 30 µm and 50 µm, respectively. For the same experiment time (8 h), only an area of 3.45 mm × 3.45 mm comprising 69 × 69 pixels could be measured since a long dwell time of 5 s was needed for good counting statistics and the overhead time was 2.4 h for scanning. Fig. 3 ▸ compares Zn images from the FXI and µXI measurements. Obviously, the latter could not reveal Zn in some areas. Similar comparisons of Fe and Ca images are shown in Figs. S3–S4. To explain the observed discrepancy, the peak-to-background ratio (PKR) of the Zn *K*_α_ signal was analyzed and is reported in Table 4 ▸. Compared with µXI, the PKB improvement of FXI is substantial for Zn, 520:40, but reversed for Ca, 7:18 — at lower photon energies, µXI becomes more efficient due to the higher photon flux of the pink beam. For Fe, FXI has a much higher signal efficiency than µXI as the former could yield similar PKB using 2720 times fewer photons per pixel.

In addition, we compare our FXI with µXI stations at the Australian Synchrotron (Howard *et al.*, 2020 ▸) and National Synchrotron Light Source II (Tappero, 2023 ▸; Linam *et al.*, 2022 ▸) as shown in Table 5 ▸. Clearly, the upside of µXI is a short dwell time per pixel (<0.5 s) attributed to their intense photon flux values; however, the downside is scan-overhead (SO) time, which can be unfavorably long for an image of enormous pixel numbers. For example, an SO time of 0.5 s pixel^−1^ requires 9 h in addition to the dwell time to cover the same pixel number as ours. In this case, simultaneous measurement of the full-field imaging is more time-efficient.

## 4. Research samples

To evaluate the performance of the FXI technique, three different samples were measured: mineral dendrite, ancient bronze and dried fish. The incident X-ray energy was set at 10 keV, the CXC gain to 128, and the frame rate to 200 Hz. Frame numbers were varied, depending on elemental concentrations in the samples. Prior to sample measurement, Cu plate data were acquired for beam intensity normalization.

**Figure 4:**
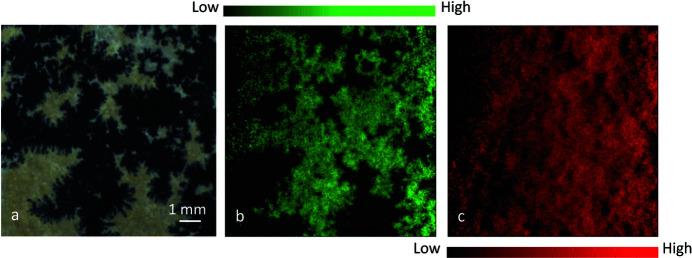
Photograph of the dendrite sample (*a*) and its Mn (*b*) and Ca (*c*) images from the FXI measurements.

Dendrite is a type of manganese oxide mineral that forms characteristic three-dimensional branching patterns in various rock types as a result of Mn-rich water percolating rock fissures. Shown in Fig. 4 ▸ is a photograph of the sample, which is black dendrite grown on limestone. The FXI data were acquired with 4.68 Mf (6.5 h), and only Ca and Mn were observed in the full-frame spectrum. The FXI data were processed as described in Section 2. Fig. 4 ▸ displays the resultant Ca and Mn images of the dendrite sample. Obviously, the Mn distribution is spatially correlated with the black branching pattern. On the other hand, Ca was mostly mapped on the whole sample, identifying limestone as a bedrock. Because of considerable sample roughness, there are certainly unexposed regions (shadowing effects), *e.g*. those shown as black (no signal) in both Ca and Mn images.

**Figure 5:**
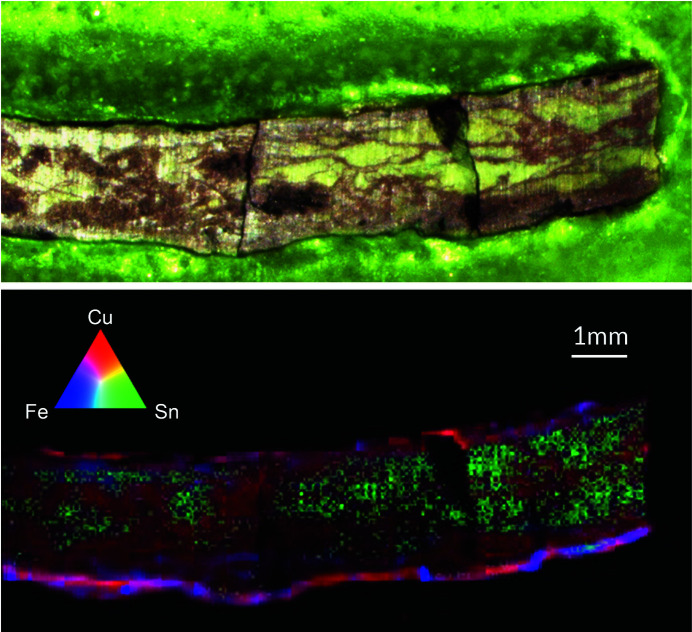
Photography of the cross-sectioned bronze sample (top) and RBG image from the FXI measurements (bottom). Copper in red, tin in green and iron in blue.

Fig. 5 ▸ shows a tricolored XRF image of a cross-sectioned ancient bronze fragment excavated from Noan Pa Cha Kao archeological site (Nakorn Ratchasima, Thailand) dating back to between 400 BC and 1000 AD. Its bronze composition was previously determined by XRF analysis, being 62–70 wt% Cu, 29–35 wt% Sn and 0.6–3.0 wt% Fe. Additionally, Cu oxide, Cu chloride and Sn oxide were identified by X-ray near-edge absorption fine-structure spectroscopy as major corrosion products from its burial environment. In this experiment, FXI data of the bronze sample were acquired with 5.76 Mf (8 h). The obtained XRF image displays high corrosion effects causing de-alloying: Sn (shown in green) considerably separates from Cu (red). Further, the Sn distribution indicates Sn leaching from the corroded bronze. On the other hand, Fe (pink) mostly remains localized with Cu.

**Figure 6:**
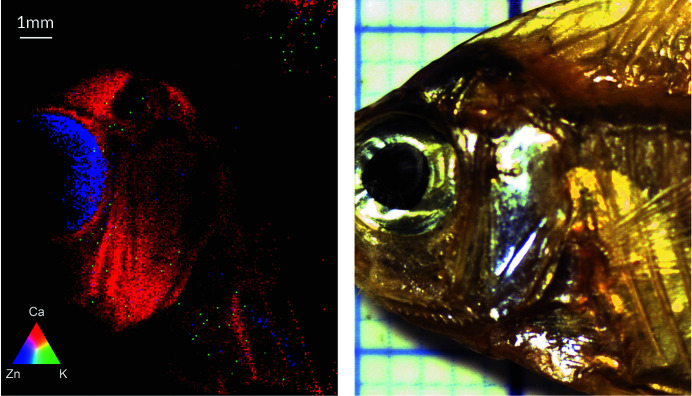
Photography of the dried fish (right) and RBG image from the FXI measurements (left). Calcium in red, potassium in green and zinc in blue.

Dried small fish are common foods in Southeast Asia, considered as a cheap source of calcium which is abundant in fish bones. A small, dried gouramis fish was selected as the FXI sample. Because of its bulging shape, only some flat area of the fish could be investigated. FXI data were acquired with 6.48 Mf (9 h). Fig. 6 ▸ shows a tricolored XRF image of the fish displaying K, Ca and Zn distributions, shown in green, red and blue, respectively. Although the K signal was too weak due to strong air absorption inside the polycapillary optics, it is still visible. The clearest elemental images were obtained for Ca located in the fish bones and Zn in the fish-eye choroid. It should be noted that a similar Zn image was reported for a fresh, rasbora fish by Ordavo *et al.* (2011 ▸) using a similar FXI set-up.

## 5. Conclusions

A full-field X-ray fluorescence imaging (FXI) station was developed at the BL8 beamline of Synchrotron Light Research Institute (SLRI), Thailand. An incident synchrotron X-ray beam was monochromated at 10 keV and has a moderate photon flux of 10^9^ photons s^−1^ (100 mA)^−1^. The experimental beam size was 13 mm × 13 mm. An energy-dispersive pnCCD array detector equipped with a polycapillary optics was employed, providing a full-frame image size of 12.3 mm × 12.3 mm comprising 256 × 256 detector pixels. A three-axis motorized stage was used for sample alignment. Image processing was carried out offline using an in-house MATLAB program. Prior to measuring a sample, a large Cu plate was measured to obtain a non-uniform incident beam profile required for normalizing pixel intensity values.

Performance evaluation of the new FXI station was carried out with various test samples. The experimental spatial resolution was determined to be 68 µm using a Cu plate and wire. The single-pixel detection limit was determined to decrease from 3.9 to 0.2 wt% for K to Zn, respectively. In terms of XRF imaging quality, peak-to-background ratios in the range 7:1 (for K) to 250:1 (for Zn) were achieved and were comparable with those of the scanning XRF station at the BL6 beamline of SLRI. However, the FXI technique was more practical since a much larger image area could be recorded for an equal time. Finally, three demonstrative samples were examined: mineral dendrite, ancient bronze and dried fish. Interesting results were obtained as follows. The dendrite image shows Mn distribution on black branching mineral and Ca distribution on white limestone. The bronze image shows de-alloyed regions of Sn and Cu and leaching of Sn from the highly corroded bronze. Lastly, the Zn image shows Zn distribution on the choroid ring around the retina of the dried fish. Therefore, we have achieved satisfactory performance of the FXI station and demonstrated possible applications of the technique.

## Supplementary Material

Figures S1 to S4. DOI: 10.1107/S1600577523002722/rv5167sup1.pdf

## Figures and Tables

**Table 1:** Technical specifications of the FXI detector used at BL8

pnCCD type	High-speed three-phase back-illuminated
Pixel size	48 µm × 48 µm
Number of pixels	65536
Image area	12.3 mm × 12.3 mm
Sensitive depth	450 µm
Frame rate	100–1000 Hz
Quantum efficiency	>95% at 3–10 keV; >30% at 20 keV
Readout noise (r.m.s.)	<3 e^−^per pixel
Charge transfer efficiency	>0.99995

**Table 2:** Experimental spatial resolution determined from thin Cu foil and Cu wire

	Cu foil	Cu wire, 25 µm	Cu wire, 50 µm
Transversal (µm)	66	69	65
Longitudinal (µm)	68	70	71

**Table 3:** Average pixel intensity, background and analyzed pixel detection limits (PDLs)

	K	Ca	Ti	V	Cr	Mn	Fe	Co	Ni	Cu	Zn
Net intensity (×10^−5^ cps)	2	7	19	28	40	31	27	54	86	42	104
Background (×10^−8^ cps)	6	11	3	6	8	8	11	25	33	22	16
PDLs (wt%)	3.9	1.6	0.3	0.7	0.6	0.4	0.6	0.4	0.3	0.6	0.2

**Table 4:** Peak-to-background (PKB) values of the C2 images obtained from the µXI and FXI stations at SLRI and ratios of incident photons per pixel, µXI to FXI

	Ca	Fe	Zn
µXI PKB	18	150	40
FXI PKB	7	140	520
Photon-in ratio	2340	2720	390

**Table 5:** Technical information of the XRF imaging stations at SLRI, Australian Synchrotron (ANSTO) and National Synchrotron Light Source (NSLS-II) BM, IVU and WG stand for bending magnet, in-vacuum undulator and wiggler, respectively.

	SLRI	SLRI	ANSTO	NSLS-II
Beamline	BL8	BL6b	XFM	XFM
X-ray source	BM	BM	IVU	WG
Beam size (µm)	13000 × 2000†	30–50	2, >100	2–10
Resolution (µm)	67	50	2, >100	2–10
Photon flux per pixel (photons s^−1^)	3 × 10^4^	3 × 10^10^ to 2 × 10^12^	10^9^ to 10^11^	∼10^13^
Dwell time per pixel (s)	>620‡	5	0.5–2	0.1–0.5
Scan overhead per pixel (s)	0	1.8	0.5	0.002–2
Detector model	CXC	Vortex EM-90	Maia Rev C, Vortex EM-90	Canberra 7SDD
Readout noise	<3 e^−^			

† Normal incidence.

‡ For XRF energy < Fe *K*_α_ (6.4 keV).
