# Supplementary material for: A new full-field XRF imaging station at Synchrotron Light Research Institute
Source: J Synchrotron Radiat. 2023 Apr 24;30(Pt 3):655–61. doi: 10.1107/S1600577523002722 (PMC10161892; doi:10.1107/S1600577523002722)
Supplement: Supplementary file 1 [file s-30-00655-sup1.pdf]

JOURNAL OF SYNCHROTRON RADIATION

Volume 30 (2023)

Supporting information for article:

A new full-field XRF imaging station at Synchrotron Light Research Institute

Wantana Klysubun, Pongjakr Tarawarakarn, Noppawath Thamsanong, Sompin Mahakhod, Penphitcha Amonpattaratkit and Chanakan Cholsuk

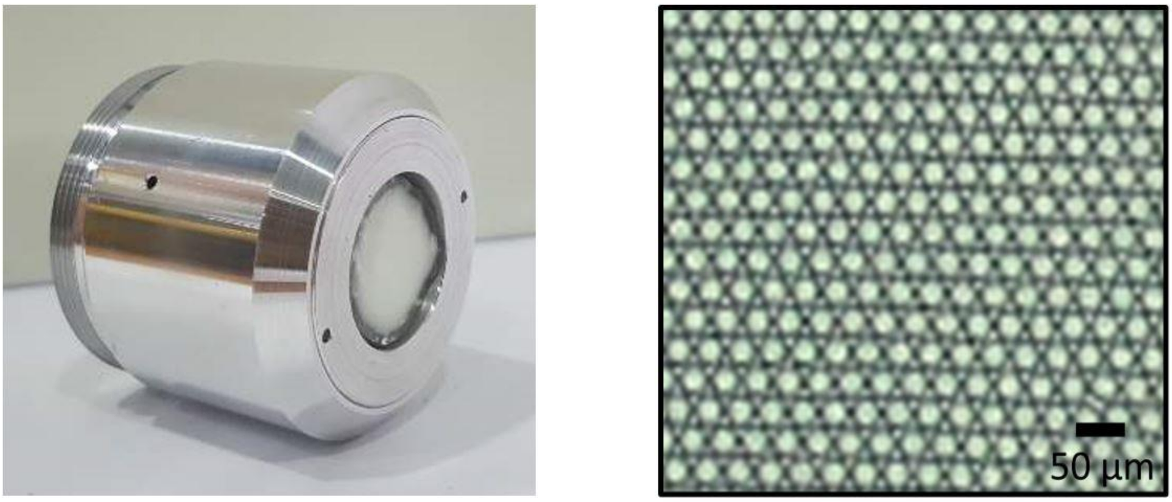

Figure S1 Photograph of the polycapillary optics (left) and magnified image of the optical channels (right).

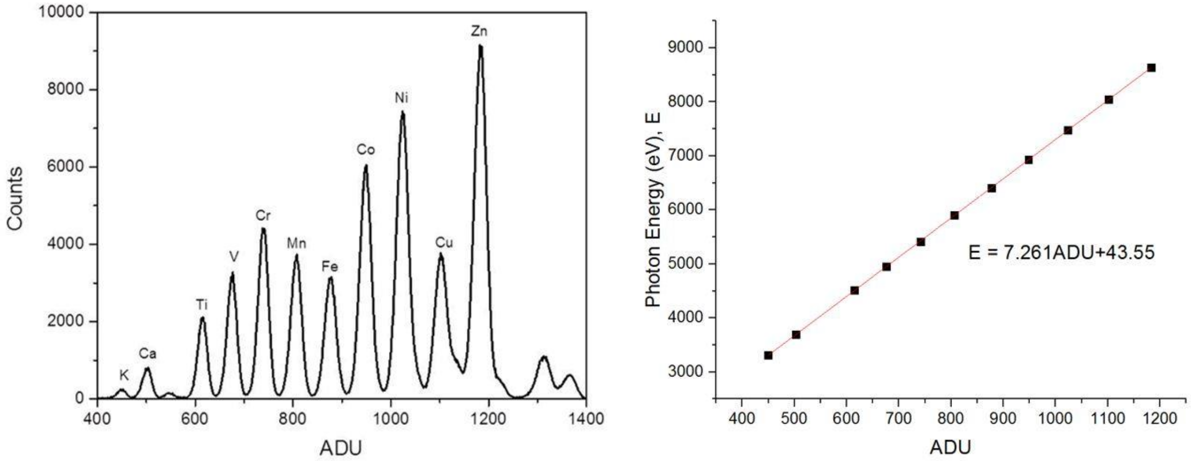

Figure S2 Full-frame XRF spectrum of the C1 sample (left) and the best fitted photon energy as a linear function of ADU (right).

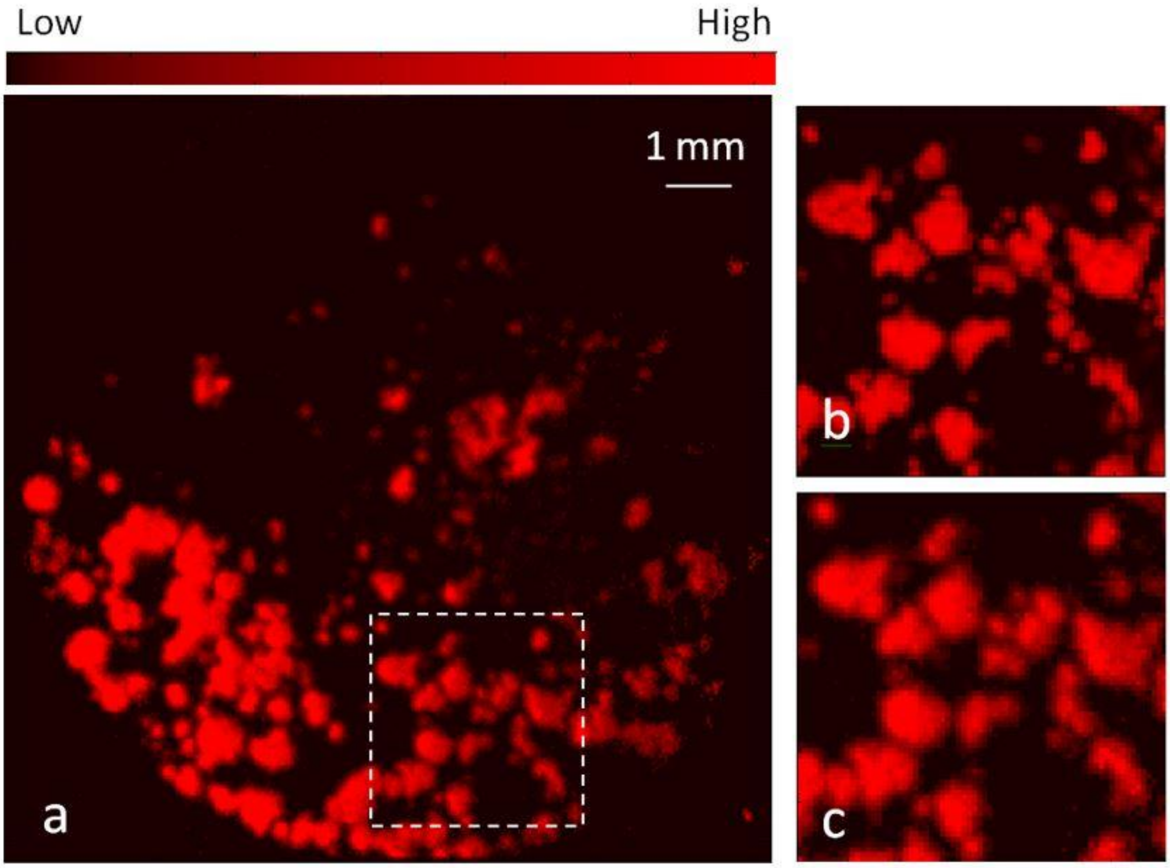

Figure S3 Fe image of the C2 sample recorded at the FXI station (a), Fe image in the dashed frame recorded at the µXI (b) and FXI (c) stations.

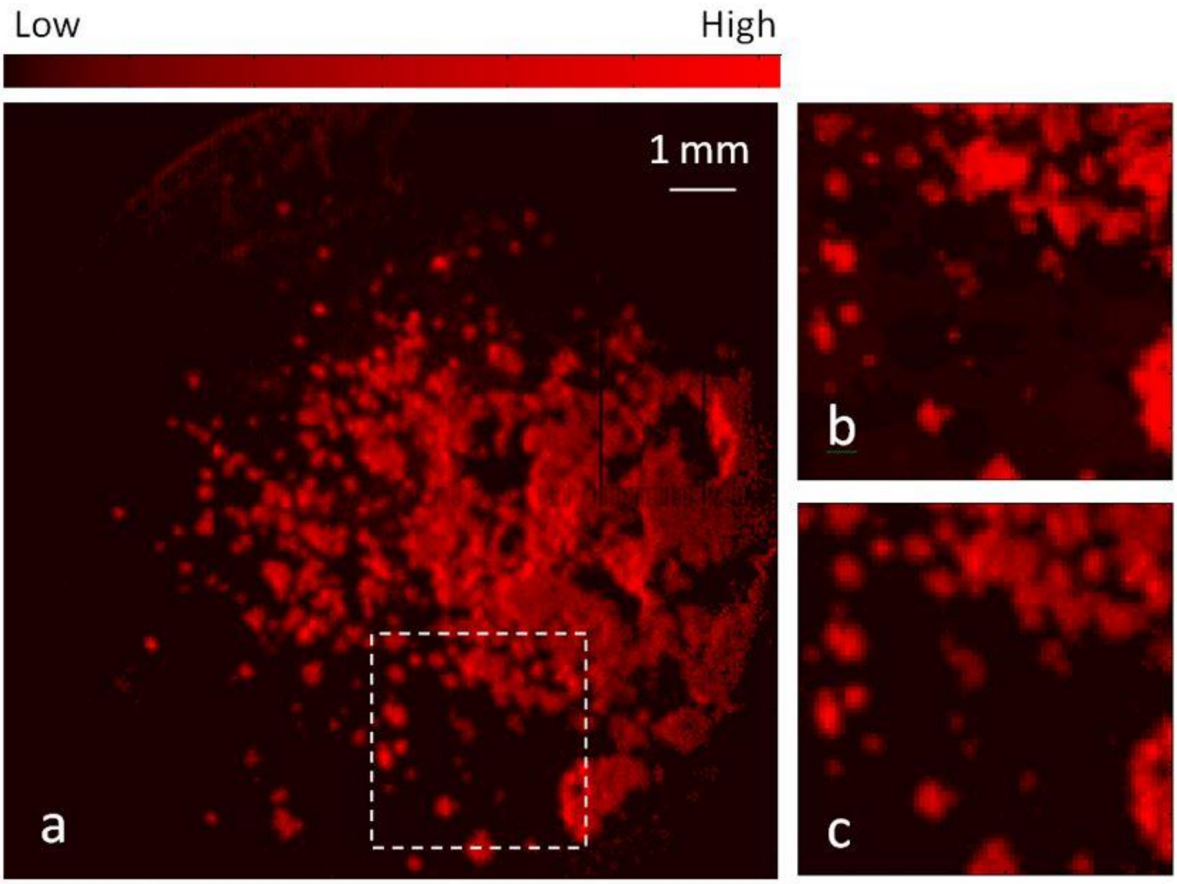

Figure S4 Ca image of the C2 sample recorded at the FXI station (a), Ca image in the dashed frame recorded at the μXI (b) and FXI (c) stations.
